# Heat-induced hyperthermia impacts the follicular fluid proteome of the periovulatory follicle in lactating dairy cows

**DOI:** 10.1371/journal.pone.0227095

**Published:** 2019-12-30

**Authors:** Louisa A. Rispoli, J. Lannett Edwards, Ky G. Pohler, Stephen Russell, Richard I. Somiari, Rebecca R. Payton, F. Neal Schrick

**Affiliations:** 1 Department of Animal Science, The University of Tennessee, Institute of Agriculture, AgResearch, Knoxville, TN, United States of America; 2 ITSI–Biosciences, LLC, Johnstown, PA, United States of America; University of Florida, UNITED STATES

## Abstract

We hypothesized that heat-induced perturbations in cumulus cells surrounding the maturing oocyte may extend to the mural granulosa of the periovulatory follicle in the heat-stressed cow to subsequently the follicular fluid proteome. Lactating Holsteins were pharmacologically stimulated to have a dominant follicle that was capable of responding to a gonadotropin releasing hormone-induced luteinizing hormone surge. Following gonadotropin releasing hormone administration, cows were maintained at ~67 temperature humidity index (THI; thermoneutral conditions) or exposed to conditions simulating an acute heat stress event (71 to 86 THI; heat stress for ~12 h). Dominant follicle collection was conducted in the periovulatory period ~16 h after gonadotropin releasing hormone. Follicular fluid proteome from thermoneutral (n = 5) and hyperthermic (n = 5) cows was evaluated by quantitative tandem mass spectrometry (nano LC-MS/MS). We identified 35 differentially-abundant proteins. Functional annotation revealed numerous immune-related proteins. Subsequent efforts revealed an increase in levels of the proinflammatory mediator bradykinin in follicular fluid (P = 0.0456) but not in serum (P = 0.9319) of hyperthermic cows. Intrafollicular increases in transferrin (negative acute phase protein) in hyperthermic cows (P = 0.0181) coincided with a tendency for levels to be increased in the circulation (P = 0.0683). Nine out of 15 cytokines evaluated were detected in follicular fluid. Heat stress increased intrafollicular interleukin 6 levels (P = 0.0160). Whether hyperthermia-induced changes in the heat-stressed cow’s follicular fluid milieu reflect changes in mural granulosa, cumulus, other cell types secretions, and/or transudative changes from circulation remains unclear. Regardless of origin, heat stress/hyperthermia related changes in the follicular fluid milieu may have an impact on components important for ovulation and competence of the cumulus-oocyte complex contained within the periovulatory follicle.

## Introduction

Greater than 70% of the world’s cattle population reside in subtropical and tropical conditions [1]. In the US, heat stress conditions can and do occur anywhere the temperature-humidity index (THI) rises above the thermal neutral zone for dairy cattle (> 71 THI [2, 3]). Heat stress related losses because of decreased milk production, increased culling, and reduced pregnancy rates cost U.S. producers approximately one billion dollars annually [4]. Elevated ambient conditions above the thermoneutral zone evoke different physiological thermoregulatory responses (e.g., panting and sweating [5, 6]) in an effort to maintain body temperature. Depending on severity and duration, hyperthermia (an increase in core body temperature above the critical point (> 39.5°C [7]) may occur. Heat-induced increases in hyperthermia during the time period of estrus (i.e., when female is sexually active and the oocyte contained within the ovulatory follicle has resumed meiosis) are especially problematic. Experimental induction in superovulated heifers, by exposing to elevated ambient temperatures for ~10 h after the onset of behavioral estrus, reduced quality of embryos resulting after artificial insemination [8].

Functional changes in the cumulus-oocyte complex and ovulatory follicle components are likely problematic. Direct exposure of cumulus-oocyte complexes to a physiologically-relevant elevated temperature during the first half of *in vitro* maturation reduces embryo development [9–12] in a manner consistent with what has been observed after heat-induced hyperthermia occurring *in vivo* near the time of estrus [8, 13, 14]. Heat stress exposure during the first half of *in vitro* maturation heightens progesterone production and alters the transcriptome and interconnectedness of the cumulus [15–17] surrounding the maturing oocyte. Heat-induced differences in cumulus function persist despite efforts to mature cumulus-oocyte complexes under thermoneutral conditions for the remainder of maturation [16]. Similar consequences may occur in cells comprising the ovulatory follicle. Acute exposure of follicular cells to heat stress conditions increased gonadotropin-stimulated progesterone secretion [18].

Taken together, we hypothesized that hyperthermia-induced perturbations in the cumulus cells enveloping the maturing oocyte may extend to the mural granulosa of the periovulatory follicle in the heat-stressed cow to alter the follicular fluid milieu. Depending on the extent to which this may be occurring, functional changes may be sufficient to explain some of the reductions in developmental competence of the heat stressed-oocyte resident within. The primary objective of this study was to characterize the proteome within the periovulatory follicle in response to heat-induced hyperthermia when the maturing oocyte is most susceptible to elevated temperatures [8, 9, 19]. To that end, we utilized quantitative tandem mass spectrometry (nano LC-MS/MS) to discern protein changes in individual follicle aspirates from hyperthermic cows compared to thermoneutral counterparts. Subsequent efforts examined levels of immune-related proteins (bradykinin and transferrin) and cytokines in follicular fluid and sera.

## Materials and methods

### Materials

Except where noted, chemicals and reagents were obtained from MilliporeSigma (St. Louis, MO, USA).

### Animals

**Fig 1:**
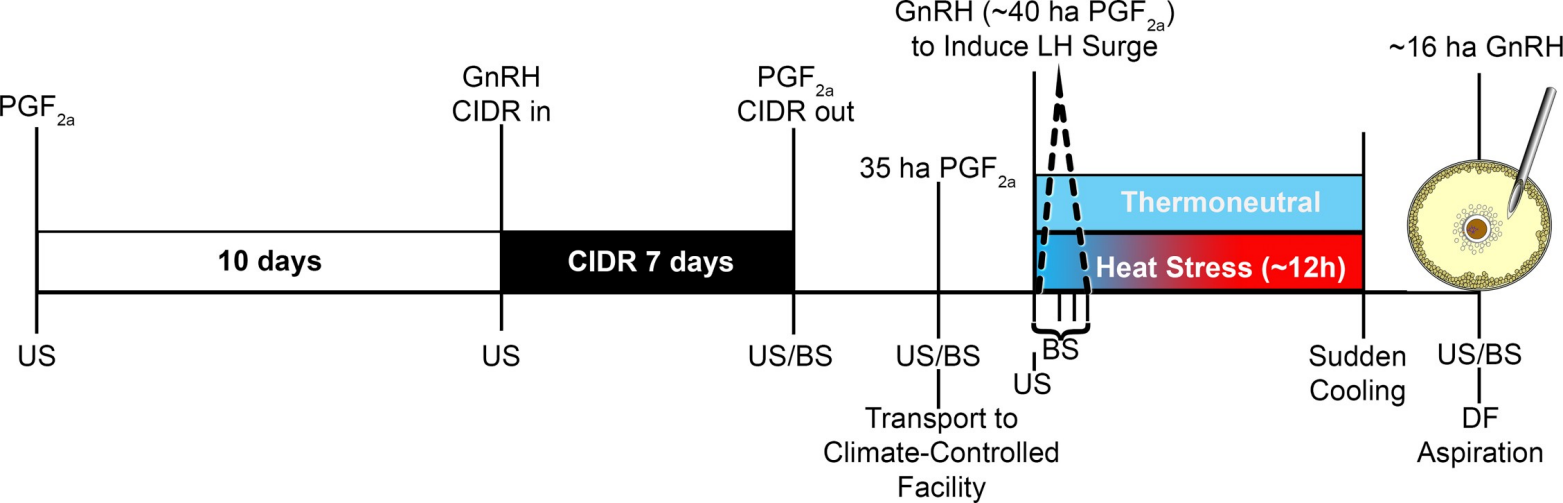
Experimental Schematic. Pharmacological protocol, treatment conditions, and timing of ultrasound guided transvaginal follicular aspiration of the ovulatory follicle in lactating dairy cows maintained at thermoneutral or heat stress conditions [20]. At time of transport (35 h after [ha] final PGF_2α_), cows were randomly allocated to either thermoneutral or heat stress conditions. For thermoneutral conditions, cows were maintained at ~67 temperature-humidity index (THI). Heat stress conditions consisted of THI being steadily increased (final THI ~83) starting within 2 h of final GnRH analog administration. After ~12 h exposure to elevated THI, heat-stressed cows were suddenly cooled. Contents of the dominant follicle (DF) were aspirated ~16 ha GnRH. Ultrasonography (US) and blood sampling (BS) were done to monitor follicle turnover and growth as well as circulating hormone levels (estradiol, progesterone and LH). CIDR = progesterone containing controlled internal drug releasing device.

Results described herein are those obtained from a subset of cows included in a larger study aimed at developing “an *in vivo* model to assess the thermoregulatory response of lactating Holsteins to an acute heat stress event occurring after a pharmacologically-induced LH surge” [20]. Animal use was approved by the University of Tennessee, Knoxville Institutional Animal Care and Use Committee. In this initial effort, and described in detail by Abbott *et al*. [20], 29 lactating, primi- and multiparous Holstein dairy cows were utilized. Pharmacological-based protocol to obtain a dominant/ovulatory follicle after inducing an endogenous luteinizing hormone (LH) surge utilized prostaglandin F_2α_ (PGF_2α_; dinoprost tromethamine, Lutalyse®; Pfizer Animal Health, Florham Park, NJ, USA), gonadotropin releasing hormone (GnRH) analogue (gonadorelin hydrochloride, Factrel®; Zoetis Inc, Kalamazoo, MI, USA), controlled intravaginal drug release (CIDR) devices containing progesterone (Eazi-breed CIDR, Pfizer Animal Health), and is depicted in Fig 1. Cows, confirmed using ultrasound to have a dominant follicle ~35 h after final PGF_2α_, were randomly allocated to treatment and transported to a climate-controlled animal facility. Approximately 40 h after final PGF_2α_, cows were given a GnRH analogue (gonadorelin diacetate tetrahydrate, Cystorelin®; Merial Inc./Boehringer Ingelheim, Duluth, GA, USA) to induce an LH surge (defined as an increase of at least 3 SD above basal LH levels occurring between 2 to 4 h post GnRH administration).

Ambient room conditions were maintained at a THI of 65.8 ± 0.1 for thermoneutral as per Abbott *et al*. [20]. The THI utilized to impose heat stress ranged from 71.0 to 86.4 with the average being 83.2 ± 0.2 to simulate conditions that may occur during an acute heat stress event on a given day. Changes in THI were initiated within 2 h of final GnRH administered to induce an LH surge. Heat stress ceased at ~12.1 ± 0.2 h after final GnRH administration; within ~15 min room was close to thermoneutral. Room environments were monitored continuously via HOBO U23 Pro v2 temperature and relative humidity data logger (accurate to ± 0.21°C and ± 2.5% RH; Onset Computer Corporation, Bourne, MA, USA). The THI was calculated based on the ambient temperature (Ta) and relative humidity (RH) using the following equation: (0.8 * Ta) + ((RH / 100) * (Ta– 14.4)) + 46.4 [21, 22]. During this time period, cows were maintained in individual pens to enable recording of rectal temperature (GLA M700 livestock thermometer accurate to ± 0.1°C; GLA Agricultural Electronics, San Luis Obispo, CA, USA) and respiration rate (flank movements observed for 15 seconds). Rectal temperature (RT) and respiration rate (RR; breaths per minute [bpm]) were recorded at least once hourly in cows maintained under thermoneutral conditions and every 15 and 30 minutes, respectively for heat-stressed cows.

To test hyperthermia-related hypotheses, nine of the 12 cows that were heat stressed by Abbott *et al*. [20] and confirmed to respond to pharmacological based protocol to induce an ovulatory follicle were retrospectively subdivided into two different groups: intermediate cows (n = 4) had increased respiration rates but only a modest increase in rectal temperature (38.4 vs 39.1 for thermoneutral and intermediate groups, respectively, Table 1); hyperthermic cows (n = 5) had higher respiration rates and rectal temperatures averaging 39.8°C; Table 1). Upon return to thermoneutral conditions after ceasing heat stress, rectal temperature and respiration rate of cows decreased by 0.012 ± 0.003°C per min and 0.6 ± 0.12 bpm in those that became hyperthermic versus 0.015 ± 0.002°C per min and 0.8 ± 0.09 bpm in those cows exhibiting an intermediate response. Rectal temperature and respiration rate were 39.0°C and 65.0 bpm for hyperthermic cows and 38.5°C and 55.5 bpm for intermediate cows 2 h later. Depending on sample size of the variable of interest to be examined herein, this allowed for comparing heat-stress related outcomes (i.e., cows that became hyperthermic or had an intermediate response) to “equivalent” thermoneutral controls (n = 5; Table 1 & S1 Fig).

**Table 1:** Characteristics of cows exposed to thermoneutral or heat-stress conditions.

Parameter	Thermoneutral	Intermediate	Hyperthermic	P-Value
N = ^1^	5	4	5	----
Respiratory rate (breaths/minute)^2^	47.9 ± 3.1^A^	101.3 ± 3.1^B^	111.9 ± 3.4^C^	< 0.0001
Rectal temperature (°C)^2^	38.4 ± 0.12^A^	39.1 ± 0.14^B^	39.8 ± 0.12^C^	< 0.0001
Parity	1.75 ± 0.6	1.67 ± 0.6	1.78 ± 0.6	0.9820
Days in milk	205 ± 40	189 ± 42	189 ± 41	0.8020
Milk produced daily (kg/d)	68.0 ± 6.8	81.8 ± 6.8	74.6 ± 5.2	0.4186

Data presented as least squares means ± SEM

^1^N = Number of cows per group.

^2^Average responses during the 12 h of being maintained at either thermoneutral or heat-stressed conditions.

^ABC^Values that do not share a letter differ significantly within a row comparison

### Ultrasound guided transvaginal aspiration of follicular fluid from ovulatory follicle

At ~16 h post GnRH (Fig 1), cows were moved to a smaller pen and given an epidural (up to 6 mL VetOne® 2% Lidocaine; MWI Veterinary Supply Co., Boise, ID, USA) to minimize rectal contractions before using transrectal ultrasonography to locate the ovary containing the periovulatory follicle. Cross-sectional diameters were recorded at this time. Then an ultrasound probe (SSD-500V 7.5 MHz linear array probe; Aloka Company Ltd., Wallingford, CT, USA) secured to a handle containing the needle guide was inserted into the vagina and situated against the ovary containing the periovulatory follicle. The aspiration needle (3-inch, 18 gauge) was gently pushed through the vaginal wall and positioned to penetrate the ovarian stroma and periovulatory follicle. Follicular fluid was aspirated into a syringe, microscopically examined for presence of cumulus-oocyte complex and then processed for hormone and proteomic analyses (see below). The syringe was replaced and the collapsed follicle was flushed 3 to 4 times with HEPES-TL [23] containing 1% polyvinyl alcohol and 10,000 U/L heparin. Cumulus-oocyte complexes and granulosa cells recovered from follicular fluid/flush media were utilized in companion RNA-sequencing studies beyond scope of studies described herein.

### Sample processing and hormone analysis

Whole blood was collected via coccygeal vein or artery into 10 mL vacutainer serum separation tubes (Becton, Dickinson and Company; Franklin Lakes, NJ, USA) and allowed to clot on ice for ~4 h. Tubes were warmed at room temperature for 30 minutes before centrifugation (1,000 *x g* for 20 min at room temperature). To minimize issues with multiple freeze-thaw cycles, serum was decanted into multiple tubes then stored at -80°C.

Fluid from each periovulatory follicle was collected and kept separate. Follicular fluid was centrifuged to eliminate cells and debris (2,000 *x g* for 10 min at 4°C) then divided into multiple aliquots and snap-frozen in liquid nitrogen prior to storage at -80°C. Total protein content was evaluated using the FluoroProfile^TM^ protein quantification kit before submitting samples to ITSI-Biosciences (Johnstown, PA, USA) for proteomic profiling.

Levels of progesterone in serum and follicular fluid were assessed using the ImmuChem progesterone double antibody radioimmunoassay kit (MP Biomedicals, Costa Mesa, CA, USA) according to manufacturer specifications and previously validated by Pohler et al. [24]. Follicular fluid was diluted either 1:5 or 1:20 to ensure within detection limits of assay. Intra- and inter-assay coefficients of variation for the progesterone RIA were 1.61 and 4.06%, respectively. Serum was extracted and concentrations of estradiol measured by RIA as described previously [25]. Intra-assay coefficient of variation for the estradiol RIA was 3.36%. Follicular fluid estradiol was diluted 1:2,500 and determined using the DetectX® serum 17β-estradiol multi-species enzyme immunoassay (Arbor Assays^TM^, Ann Arbor, MI, USA) as per manufacturer’s instructions. Intra-assay coefficient of variation for the estradiol EIA was 3.20%.

### Proteomic profiling of follicular fluid

Ten individual follicular fluid aspirates (n = 5 thermoneutral and n = 5 hyperthermic cows) were submitted for proteomic profiling. Only follicular fluid samples without macroscopic blood contamination and an estradiol:progesterone ratio < 2 [26, 27] were used for proteomic profiling. Sample preparation, quantitative tandem mass spectrometry, and protein identification were performed by ITSI-Biosciences as follows.

After determining protein concentration (ToPA Bradford Protein Assay Kit; ITSI-Biosciences), fifty micrograms of each sample was diluted using 100 mM triethylammonium bicarbonate buffer (TEAB) with 1% SDS before reduction, alkylation and precipitation (Total Protein Precipitation kit; ToPREP, ITSI-Biosciences) to remove interfering substances. Precipitated proteins were re-suspended in 200 mM TEAB and subjected to an overnight trypsin digestion. Digested samples were individually labeled with TMT 10-plex isobaric protein labelling reagent set (Thermo Fisher Scientific; Waltham, MA, USA) according to manufacturer’s protocol. The ten labeled samples were combined into a single multiplex sample then subjected to fractionation by strong cation-exchange chromatography (SCX) and the fractions eluted with 50 mM, 150 mM, 250 mM, 350 mM and 450 mM ammonium acetate were collected. All the SCX fractions were desalted using a C18 cartridge (Glygen Corp., Columbia, MD, USA), dried under vacuum and then re-suspended in 2% acetonitrile/0.1% formic acid solution prior to nano LC-MS/MS analysis.

Each SCX fraction was subjected to in-line reverse phase chromatography and tandem mass spectrometry utilizing an Orbitrap Fusion Lumos mass spectrometer (Thermo Fisher Scientific) in conjunction with a Thermo Scientific RSLC-nano system. Briefly, peptides were eluted from the C18 nano column using a flow rate of 200 nL/min and a liner acetonitrile gradient from 2 to 80% acetonitrile over 180 minutes followed by high and low organic washes into the mass spectrometer via a nanospray source. The spray voltage was set to 1.8 kV and ion transfer capillary was set to 275°C. A multinotch MS3 method was used where a full MS scan from m/z 350–1600 was followed by MS2 scans on the 10 most abundant ions. Data dependent MS/MS data was collected using collision induced dissociation (CID) energy of 35%, at a scan range of 400–1200 m/z. The MS3 precursors were fragmented by higher energy collision dissociation (HCD) of 65% with a scan range of 100–500 m/z. MS1 Automatic Gain Control (AGC) was set to 5e5 with an ion trap time of 50 ms. The MS2 AGC was set at 1e4 with 50 ms ion trap time, whereas the MS3 AGC was set at 1e5 with an ion trap time of 105 ms. Unassigned charge states and charge states of +1 and > +6 were excluded for MS/MS selection. A dynamic exclusion of 60 s was set. The mass spectrometry proteomics data have been deposited to the ProteomeXchange Consortium via the PRIDE [28] partner repository with the dataset identifier PXD015735 and 10.6019/PXD015735.

Data extracted after LC-MS/MS analyses (m/z ratios; .raw data files) were searched against the *Bos taurus* (Bovine) database (downloaded November 27, 2017) from the Universal Protein Resource [29] using the multidimensional protein identification technology (MudPIT) option and the SEQUEST HT algorithm in Proteome Discoverer 2.2 (Thermo Fisher Scientific). Trypsin was the selected enzyme allowing for up to two missed cleavages per peptide. Carbamidomethyl of cysteine, N-terminal TMT6-plex and Lysine TMT6-plex were used as static modifications whereas oxidation of methionine was used as a variable modification. Proteins were identified when one or more unique peptides have X-correlation scores greater than 1.5, 2.0, and 2.5 for respective charge states of +1, +2, and +3. For quantitation, the signals of the reporter ions of each MS/MS spectrum were used to automatically calculate the relative abundance (ratio) of the peptide(s) identified in each spectrum. To determine confidence, the Percolator algorithm was used for peptide spectrum matches (PSMs) validation. A false discovery rate (FDR) threshold of < 1% was utilized in Proteome Discoverer 2.2 (Thermo Fisher Scientific) to select high confidence peptides to use for identification of proteins.

### Immune-related proteins in follicular fluid and sera

To validate some of the results of the proteomic analysis, follicular fluid and sera collected from the thermoneutral, intermediate and hyperthermic cows were evaluated. Evaluated sera included samples collected at time of GnRH administration (~40 h after PGF_2α_) to induce an LH surge and at time of dominant follicle aspiration (~16 h after GnRH; Fig 1). To examine the functioning of the kininogen-kallikrein system, a bradykinin ELISA (Enzo Life Sciences, Farmingdale, NY, USA) with a sensitivity of 24.8 pg/mL was utilized as per manufacturer instructions. Follicular fluid and sera were diluted 1:16 with provided solution and measured in duplicate. Intra- and inter-assay coefficients of variation for bradykinin ELISA were 9.13 and 11.11%, respectively. Levels of transferrin were quantified using a double antibody sandwich ELISA (Innovative Research, Inc, Novi, MI, USA) with bovine transferrin calibrator (standard curve range 18.75–600 ng/mL) according to manufacturer recommendations. Follicular fluid and sera were diluted 1:40,000 with provided solution and measured in duplicate. Intra- and inter-assay coefficients of variation for transferrin ELISA were 3.77 and 4.31%, respectively.

### Cytokines in follicular fluid and sera

We utilized a Milliplex custom bovine 15-plex cytokine panel to evaluate follicular fluid from thermoneutral, intermediate and hyperthermic cows. Only sera collected from thermoneutral and hyperthermic animals were available for assessment. Sera evaluated included samples collected at time of GnRH administration (~40 h after PGF_2α_) to induce LH surge and at time of dominant follicle aspiration (~16 h after GnRH; Fig 1). The assay was performed according to manufacturer instructions with standards and samples in duplicate to measure C-C motif chemokine ligand 2 (CCL2), C-C motif chemokine ligand 3 (CCL3), C-C motif chemokine ligand 4 (CCL4), interleukin 1α (IL-1α), interleukin 1β (IL-1β), interleukin 1 receptor antagonist (IL-1RA), interleukin 2 (IL-2), interleukin 4 (IL-4), interleukin 6 (IL-6), interleukin 8 (IL-8), interleukin 10 (IL-10), interleukin 17A (IL-17A), interferon γ (IFNγ), interferon γ-induced protein 10 (IP-10), and tumor necrosis factor α (TNFα). Data were acquired on a validated and calibrated Luminex 200 (Luminex, Austin, TX, USA) with a detection target of 50 beads per region and recommended doublet discriminator gates of 5,000 to 8,000. Milliplex Analyst 5.1 software (MilliporeSigma) was then used to plot standard data using five parameter regression with default automated best curve fit and weighting (all fitted to ≥ 6 points). Resulting detection limits and intra-assay coefficient of variations are presented in Table 2.

**Table 2:** Detection limits and precision of custom bovine 15-plex cytokine panel.

	Limits of Detection^1^		
	Lower (pg/mL)	Upper (pg/mL)	Intra-assay CV
C-C motif Chemokine Ligand 2 (CCL2)	3.58	26,823	11.2%
C-C motif Chemokine Ligand 3 (CCL3)	11.03	783,911	6.7%
C-C motif Chemokine Ligand 4 (CCL4)	0.77	193,610	5.6%
Interleukin 1α (IL-1α)	1.09	2,036	7.0%
Interleukin 1β (IL-1β)	1.31	56,735	5.5%
Interleukin 1 Receptor Antagonist (IL-1RA)	11.71	199,091	4.0%
Interleukin 2 (IL-2)	3.99	518,655	6.3%
Interleukin 4 (IL-4)	3.80	101,994	6.4%
Interleukin 6 (IL-6)	0.39	14,090	5.0%
Interleukin 8 (IL-8)	6.35	21,748	7.4%
Interleukin 10 (IL-10)	1.67	95,333	5.6%
Interleukin 17A (IL-17A)	0.0032	5,848	4.1%
Interferon γ (IFNγ)	0.00085	4,047	5.0%
Interferon γ-Induced Protein 10 (IP-10)	2.42	38,751	6.1%
Tumor Necrosis Factor α (TNFα)	2.51	899,240	6.0%

CV, coefficient of variation

^1^Values derived from plots of standard data (≥ 6 points) using 5 parameter regression with automated best curve fit and weighting (Milliplex Analyst 5.1 software; MilliporeSigma)

### Statistical and bioinformatic analyses

Results are presented as least squares means ± standard error of the mean (SEM) with differences deemed significant at P ≤ 0.05 and tendency towards significance at 0.05 < P ≤ 0.1. Mean differences, unless otherwise noted, were determined using F-protected least significant differences. Thermoregulatory responses (rectal temperature and respiration rate) and physiological characteristics (parity, days in lactation, milk yield) of cows were analyzed as a randomized block design with fixed effect of response groups (thermoneutral, intermediate and hyperthermic) using generalized linear mixed models (PROC GLIMMIX, SAS 9.4, SAS Institute, Cary, NC, USA) with blocking on cow. Analysis on the data from periovulatory follicles (time of aspiration, diameter, estradiol, progesterone and protein content) was performed as a randomized block design using the fixed effect of response groups (thermoneutral, intermediate and hyperthermic) with blocking on date of experiment.

All proteins identified with high confidence peptides from LC-MS/MS analyses of follicular fluid aspirates were fully annotated and gene ontology classified using Proteome Discoverer 2.2 as previously described [30, 31]. Principal component analysis (PCA) using singular value decomposition method with unit variance scaling and hierarchical clustering using Euclidian algorithm for dissimilarity with Ward’s linkage were performed on normalized protein abundances using ClustVis (ver. 19-Feb-18 [32]). Significant changes in protein abundances were determined using Student’s t-test on log-transformed TMT ratios of proteins with the threshold for differential abundance set at P < 0.1 and combined protein FDR confidence set at high in Proteome Discoverer 2.2. Fold changes were calculated as ratio of arithmetic means in normalized abundance of hyperthermic versus thermoneutral proteins. Functional enrichment clustering was generated using the Database for Annotation, Visualization and Integrated Discovery (DAVID, ver. 6.8, Laboratory of Human Retrovirology and Immunoinformatics, Federick, MD, USA) selecting default settings for annotation categories. Functional categories and pathways were considered overrepresented when P ≤ 0.1 (Benjamini corrected) and the enrichment score was ≥ 1.3. Pathway enrichment analysis were performed with Reactome (ver. 63 [33, 34]) utilizing a threshold of P ≤ 0.1 (FDR adjusted).

Levels of bradykinin, transferrin and cytokines within follicular fluid (expressed as per milligram of total protein) and sera samples (expressed on a per milliliter basis) were analyzed as a randomized block design. Model included the fixed effect of response (thermoneutral, hyperthermic and intermediate) by time of collection where appropriate (at final GnRH or at dominant follicle aspiration) with blocking on date of experiment and cow. Cytokine values below the limit of detection (LOD) were substituted with the cytokine specific LOD divided by square root of 2. In instances where values were greater than detection limit (DL), value was set at cytokine specific DL. Normality of data was evaluated using Shapiro-Wilks test and removal of outliers was implemented when required to achieve normal distribution (W > 0.9).

## Results

### Periovulatory follicle characteristics at time of aspiration

Contents of the periovulatory follicle from thermoneutral, intermediate and hyperthermic cows were aspirated at similar times post GnRH from follicles of similar size (P > 0.2; Table 3). Estradiol, progesterone and total protein levels in follicular fluid aspirates did not differ between groups (P > 0.2; Table 3).

**Table 3:** Characteristics of the periovulatory follicle and aspirated fluid.

Parameter	Thermoneutral	Intermediate	Hyperthermic	P-Value
Time of aspiration (h)^1^	16.45 ± 0.41	16.64 ± 0.47	16.14 ± 0.44	0.5195
Follicle diameter (mm)^2^	17.73 ± 1.00	14.68 ± 1.11	16.83 ± 1.00	0.1880
Estradiol (ng/mL)	109.14 ± 11.23	76.98 ± 13.1	90.55 ± 12.3	0.1631
Progesterone (ng/mL)	74.45 ± 9.2	55.54 ± 10.5	66.48 ± 9.9	0.3063
E2:P4^3^	1.57 ± 0.19	1.34 ± 0.22	1.44 ± 0.19	0.7333
Total protein (mg/mL)^4^	42.05 ± 3.9	32.87 ± 4.5	35.80 ± 4.2	0.1791

Data presented as least squares means ± SEM

^1^Number of hours after GnRH administration

^2^Average cross-sectional diameter at time of dominant follicle aspiration

^3^Estradiol to progesterone ratio

^4^Values obtained with the FluoroProfile^TM^ protein quantification kit

### Proteomic profile of follicular fluid after LH surge

**Fig 2:**
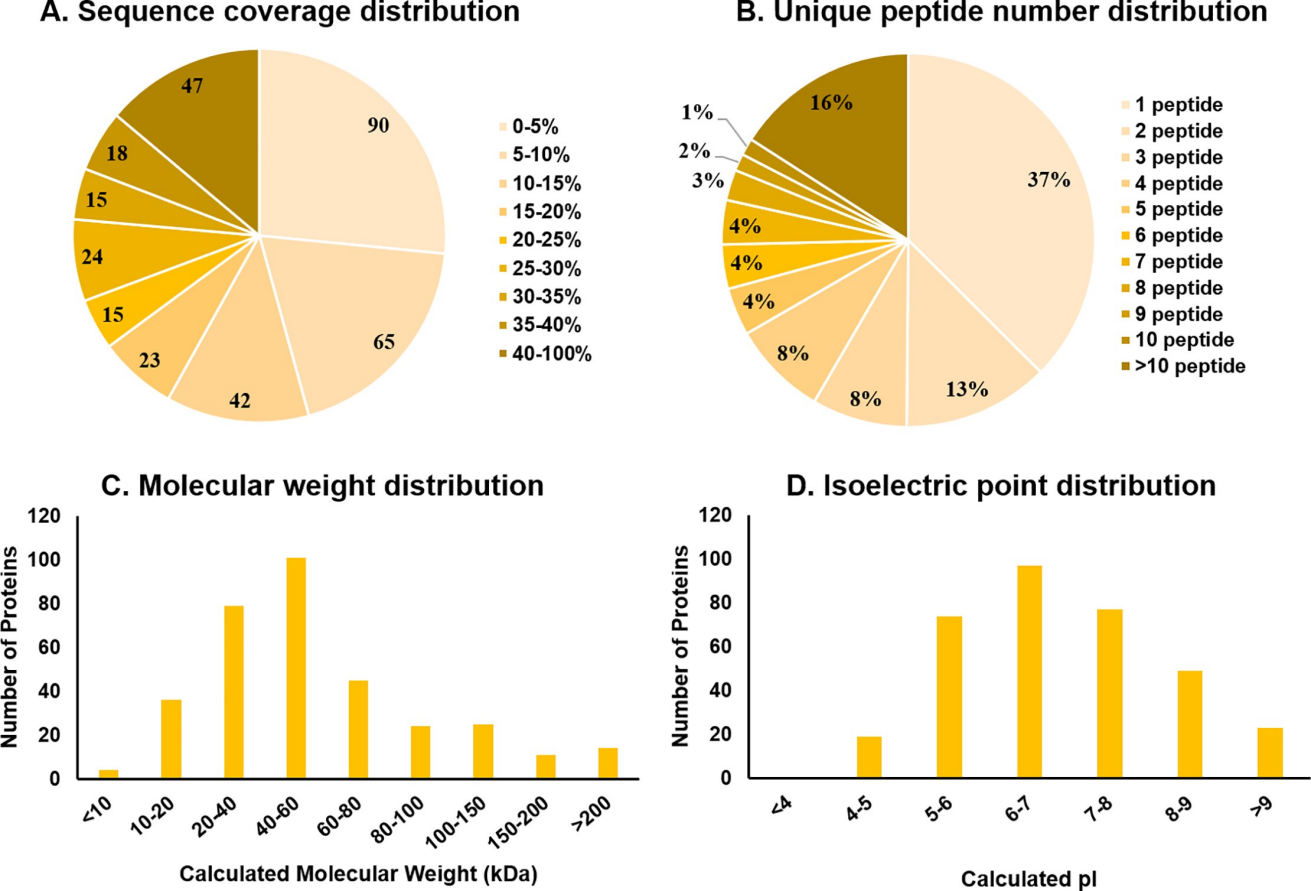
Characteristics of peptides identified within follicular fluid. The distribution of percent of sequence coverage by identified peptides (A), number of peptide sequences unique to a protein (B), calculated molecular weight (C) and theoretically calculated isoelectric point (D) for the 339 proteins identified in follicular fluid.

The 9,822 MS/MS spectra derived from a single multiplex sample (combined from 10 individual follicle fluid aspirates) were matched to 1,910 unique peptides and resulted in identification of 339 high confidence proteins (S1 File). The amount of the protein sequence covered by identified peptides averaged at 19% (Fig 2A). For more than half of the identified proteins the sequence coverage was 10% or greater (184/339; Fig 2A) and had more than one unique peptide attributed (212/339; Fig 2B). The mass range distribution of the proteins identified spanned from 5 kDa to 550 kDa with most weighing between 10 and 80 kDa (79%, Fig 2C). The isoelectric point for the majority of proteins (320/339) was greater than five (Fig 2D).

**Fig 3:**
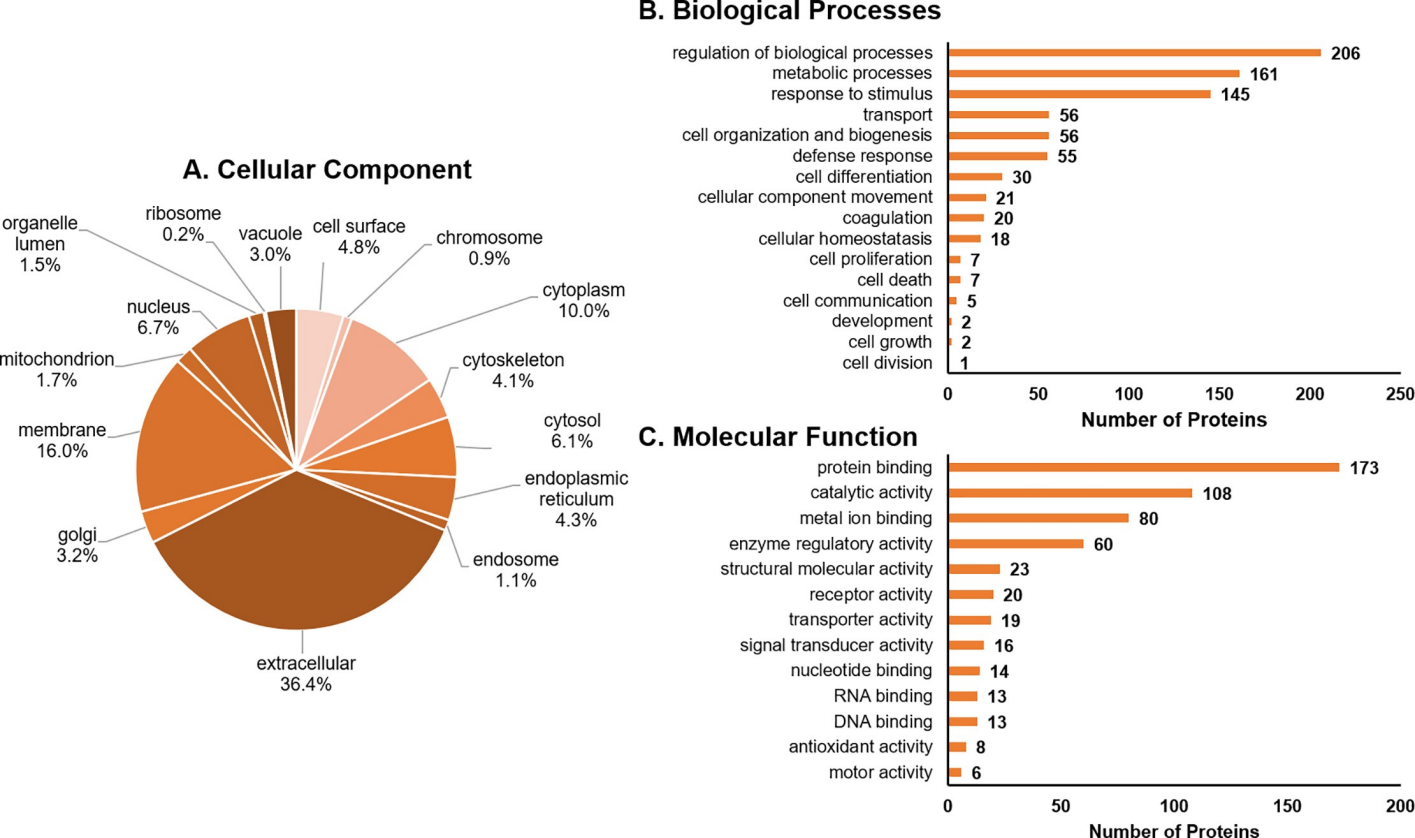
Gene ontology classification for the proteins identified in follicular fluid. Cellular component (A), biological processes (B) and molecular function (C).

Classification according to gene ontology (cellular component, biological processes, and molecular function) was available for 291 of the identified proteins. More than one-third of identified proteins were annotated with localization to extracellular component (Fig 3A). Majority of the annotated proteins classified to categories of regulation of biological processes (24%), metabolic process (19%), or response to stimulus (17%; Fig 3B). The most represented molecular functions in the proteome in follicular fluid included interaction of a molecule with one or more specific sites on another molecule (GO:0005488: protein binding) and catalysis of a biochemical reaction at physiological temperatures (GO:0003824: catalytic activity; Fig 3C).

**Fig 4:**
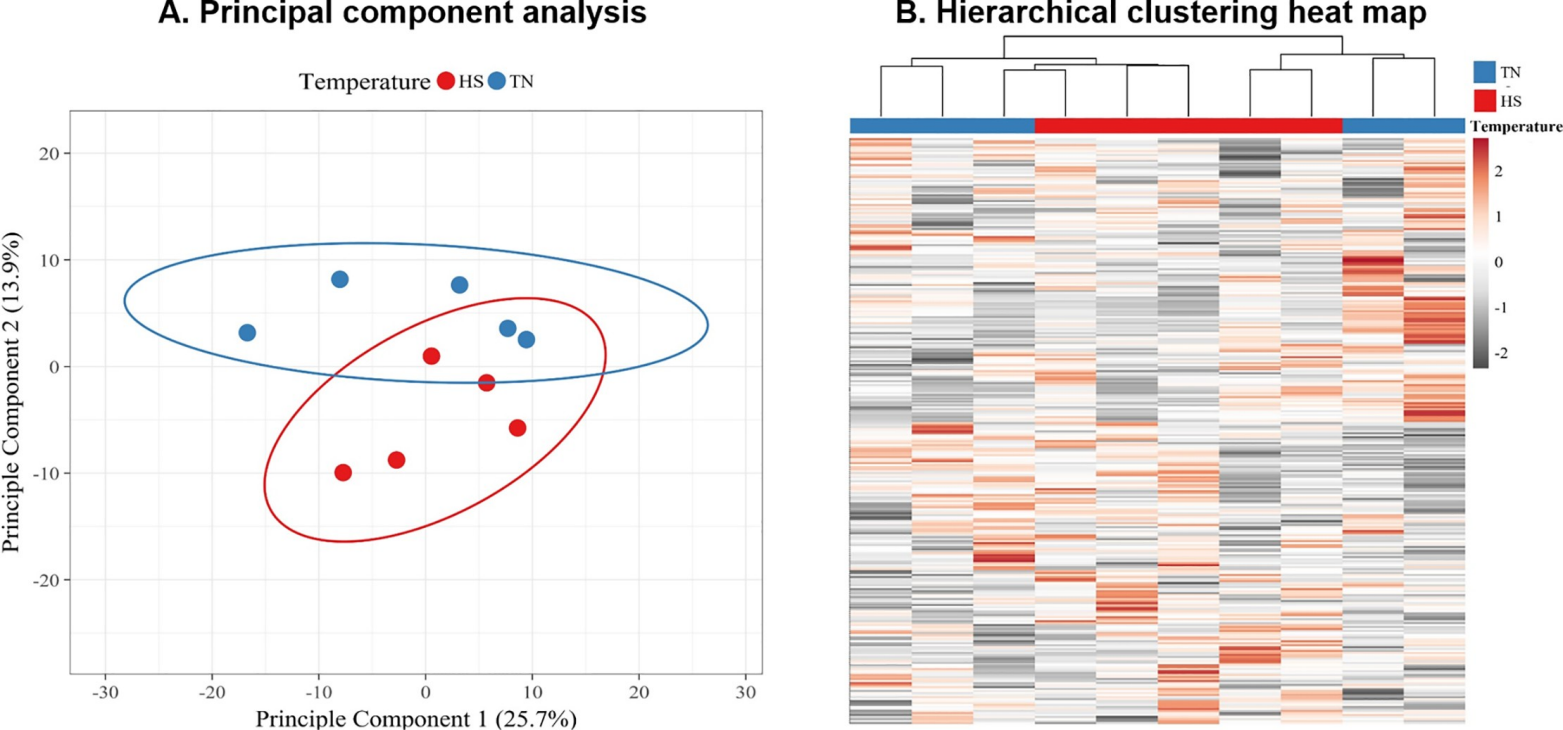
Principal component analyses and hierarchical clustering of proteins identified in follicular fluid. Principal component (PC) analysis plot (A) using singular value decomposition to calculate variance in the follicular fluid (FF) proteome collected at ~16 h post GnRH from thermoneutral (exposed to thermoneutral conditions; TN) and hyperthermic (exposed to heat stress conditions; HS) cows. The data points refer to FF samples from individual cows with temperature conditions identified by colors in legend. Prediction ellipses indicate 95% confidence region for each temperature group. Hierarchical clustering heat map (B) depicts abundance pattern of the 339 proteins identified. The heat map indicates high (dark red), low (grey) and intermediate (white) abundances for individual proteins (rows). The columns represent FF samples from individual cows with temperature identified at top of column by colors in legend.

Principal component analysis and hierarchical clustering revealed the overall proteome in the follicular fluid from five hyperthermic cows differed to some extent from the protein profiles from the five thermoneutral counterparts (Fig 4). Specifically, the samples clustered by treatment (thermoneutral versus heat stress) conditions with some overlap between the two clusters (Fig 4A), suggesting the 12 h of heat stress exposure resulted in modest protein changes within the follicular fluid. A major source of variation is derived from differences between samples within the thermoneutral group (Fig 4).

Thirty-five proteins were identified to be differentially abundant between thermoneutral and hyperthermic samples (Table 4). About one-half of the protein differences were due to moderate changes (0.67 < Hyp/TN ratio < 1.5) in abundance compared between different environmental conditions (19/35; Table 4). Only four proteins were found to be upregulated (≥ 1.5-fold higher) in the follicular fluid of hyperthermic cows: kininogen-2, serotransferrin, serglycin and syndecan (Table 4). Proteins determined to be downregulated (≤ 1.5-fold lower) due to heat stress consist of numerous cytokeratins (2, 3, 5, 10, 17, 18 & 75), myosin 18B, histidine-rich glycoprotein, alpha-2-macroglobulin, cathespin B and pleiotrophin (Table 4). Enrichment analysis revealed four functional annotation clusters (Table 5) associated with the list of differentially abundant proteins. The first annotation cluster comprised proteins associated with the following keywords: secreted, glycoprotein, signal and disulfide bond. The second annotation cluster related to enrichment in proteins for structure molecule activity, specifically intermediate filament and keratins. The third cluster contained over-representation of proteins involved in complement and coagulation cascades. Finally, in the fourth clustering were enzyme related keywords, including zymogen, hydrolase and peptidases. A query of the Reactome database yielded over-representation for numerous pathways including the regulation of IGF transport and uptake by IGF binding proteins, keratinization, lipid transport (i.e., chylomicron assembly) and complement cascade (Table 6).

**Table 4:** Differentially-abundant proteins in follicular fluid from hyperthermic versus thermoneutral cows.

Protein Description	Accession^1^	Gene Name	P-value^2^	Ratio Hyp/TN^3^	Fold Change^4^
Kininogen-2	P01045	KNG2	0.0188	14.7	14.7
Transferrin	G3X6N3	TF	0.0221	2.27	2.27
Serglycin	G5E5K5	SRGN	0.0425	1.75	1.75
Syndecan	G3MYP1	SDC4	0.1044	1.51	1.51
Beta-1,4-glucuronyltransferase 1	Q5EA01	B4GAT1	0.0797	1.32	1.32
Inhibin beta B chain	F1MVS8	INHBB	0.0104	1.3	1.3
Adiponectin	Q3Y5Z3	ADIPOQ	0.0105	1.28	1.28
Glycosylation-dependent cell adhesion molecule 1	P80195	GLYCAM1	0.0431	1.27	1.27
Alpha-2-HS-glycoprotein	B0JYN6	AHSG	0.0615	1.22	1.22
Mannan binding lectin serine peptidase 2	E1BJ49	MASP2	0.0236	1.16	1.16
Insulin-like growth factor binding protein acid labile subunit	Q09TE3	IGFALS	0.0271	1.16	1.16
Plasma kallikrein	Q2KJ63	KLKB1	0.0293	1.16	1.16
Apolipoprotein A-II	P81644	APOA2	0.0429	1.16	1.16
Carboxypeptidase B2	Q2KIG3	CPB2	0.0023	1.15	1.15
Follistatin-related protein 1	Q58D84	FSTL1	0.0654	1.14	1.14
Protein disulfide-isomerase	A6H7J6	P4HB	0.0631	1.13	1.13
Carboxypeptidase N catalytic chain	G5E5V0	CPN1	0.0716	1.06	1.06
Bleomycin hydrolase	E1BL29	BLMH	0.1028	0.87	-1.15
Collagen alpha-1(IV) chain	Q7SIB2	COL4A1	0.0716	0.86	-1.16
Insulin-like growth factor-binding protein 2	P13384	IGFBP2	0.0372	0.85	-1.17
Laminin subunit alpha 1	F1MEG3	LAMA1	0.0865	0.84	-1.19
Keratin, type I cytoskeletal 42	G3N2P6	KRT42	0.0546	0.7	-1.42
Serine protease 23	Q1LZE9	PRSS23	0.0726	0.7	-1.44
Pleiotrophin	P21782	PTN	0.0715	0.67	-1.49
Cathepsin B	P07688	CTSB	0.0859	0.65	-1.55
Keratin, type II cytoskeletal 2	G3MZ71	KRT2	0.0382	0.64	-1.57
Keratin, type II cytoskeletal 75	Q08D91	KRT75	0.0745	0.58	-1.72
Keratin, type II cytoskeletal 5	Q5XQN5	KRT5	0.0662	0.56	-1.78
Alpha-2-macroglobulin variant 5	K4JDR8	A2M	0.0685	0.52	-1.92
Keratin, type I cytoskeletal 17	A0A140T867	KRT17	0.0211	0.49	-2.02
Keratin, type I cytoskeletal 18	A6H7D3	KRT18	0.1022	0.47	-2.14
Histidine-rich glycoprotein (Factor XIIIA substrate)	Q9TS85	HRG	0.025	0.44	-2.28
Keratin, type I cytoskeletal 10	A6QNZ7	KRT10	0.0582	0.42	-2.40
Keratin, type II cytoskeletal 3	G3MXL3	KRT3	0.0576	0.39	-2.54
Myosin XVIIIB	E1BA80	MYO18B	0.0524	0.17	-5.81

^1^The Universal Protein Resource [29] identifier

^2^Student’s t-test on log-transformed TMT ratios of proteins with differential expression threshold set at P ≤ 0.1

^3^Abundance ratio of Hyperthermic (Hyp) to Thermoneutral (TN) values. Ratio > 1.5 classified as upregulated by hyperthermia, < 0.67 as downregulated by hyperthermia, values from 0.67 to 1.5 considered moderate changes.

^4^For fold change values < 1 used equation -1/ratio

**Table 5:** Functional annotation clustering of proteins identified to be altered in follicular fluid due to hyperthermia.

Annotation Cluster & Members^1^	Enrichment Score	Protein Count	P-value^2^	Associated Proteins (Gene Names)
**Cluster 1**	9.96			ADIPOQ, AHSG, A2M, APOA2, B4GAT1, CPB2, CPN1, CTSB, COL4A1, FSTL1, GLYCAM1, INHBB, IGFBP2, IGFALS, KLKB1, KNG2, LAMA1, MASP2, PTN, P4HB, PRSS23, SRGN, SDC4, TF
Secreted (UP)		16	3.8e^-10^	
Glycoprotein (UP)		16	1.1e^-08^	
Signal (UP)		22	8.2e^-09^	
Disulfide bond (UP)		18	6.8e^-09^	
**Cluster 2**	5.68			KRT2, KRT3, KRT5, KRT10, KRT17, KRT18, KRT75, LAMA1, MYO18B
Intermediate filament protein, conserved site (IP)		7	7.5e^-08^	
Intermediate filament (UP)		7	2.7e^-08^	
Intermediate filament protein (IP)		7	1.8e^-07^	
Keratin (UP)		6	3.6e^-07^	
Structural molecule activity (MF)		7	4.8e^-05^	
SMO1391:filament (SMART)		6	2.5e^-05^	
Intermediate filament (CC)		5	8.1e^-05^	
Keratin filament (CC)		4	2.0e^-03^	
Type II keratin (IP)		3	3.0e^-02^	
Keratin, type I (IP)		3	3.3e^-02^	
Coiled coil (UP)		9	2.6e^-01^	
**Cluster 3**	3.23			A2M, CBP2, KLKB1, KNG2, MASP2
Complement and coagulation cascades (KEGG)		5	3.0e^-04^	
Hemostasis (UP)		3	1.3e^-02^	
Blood coagulation (UP)		3	1.3e^-02^	
Blood coagulation (BP)		3	6.4e^-01^	
**Cluster 4**	1.98			CPB2, CPN1, CTSB, KLKB1, MASP2, PRSS23
Protease (UP)		6	2.3e^-03^	
Zymogen (UP)		3	7.8e^-02^	
Peptidase S1 (IP)		3	3.2e^-01^	
Trypsin-like cysteine/serine peptidase domain (IP)		3	3.1e^-01^	
Hydrolase (UP)		6	2.0e^-01^	
Serine-type endopeptidase activity (MF)		3	6.3e^-01^	

^1^DAVID category terms: BP–biological function, CC–cellular component, IP–Interpro, KEGG–Kyoto Encyclopedia of Genes and Genomes pathway, MF—molecular function, SMART–Simple Modular Architecture Research Tool, UP–UniProt keywords

^2^Benjamini corrected EASE score

**Table 6:** Reactome pathways over-represented in proteins identified to be altered in follicular fluid due to hyperthermia.

Pathway ID	Pathway Name	# Entities Found^1^	# Entities Total^2^	P-Value^3^	Associated Proteins (Gene Names)
R-BTA-381426	Regulation of Insulin-like Growth Factor (IGF) transport and uptake by Insulin-like Growth Factor Binding Proteins (IGFBPs)	8	146	4.38e^-05^	AHSG, APOA2, FSTL1, IGFALS, IGFBP2, P4HB, PRSS23, TF
R-BTA-8957275	Post-translational protein phosphorylation	6	128	0.0016	AHSG, APOA2, FSTL1, P4HB, PRSS23, TF
R-BTA-6809371	Formation of the cornified envelope	5	101	0.0039	KRT2, KRT3, KRT10, KRT17, KRT75
R-BTA-6805567	Keratinization	5	129	0.0089	KRT2, KRT3, KRT10, KRT17, KRT75
R-BTA-8963888	Chylomicron assembly	2	10	0.0208	APOA2, P4HB
R-BTA-8963898	Plasma lipoprotein assembly	2	16	0.0028	APOA2, P4HB
R-BTA-166658	Complement cascade	3	68	0.0564	CPB2, CPN1, MASP2

^1^Number of mapped identifiers that match pathway for selected molecular type

^2^Total number of identifiers in the pathway for selected molecular type

^3^FDR adjusted P-value, threshold set at P ≤ 0.1

### Immune-related proteins in follicular fluid and sera

**Fig 5:**
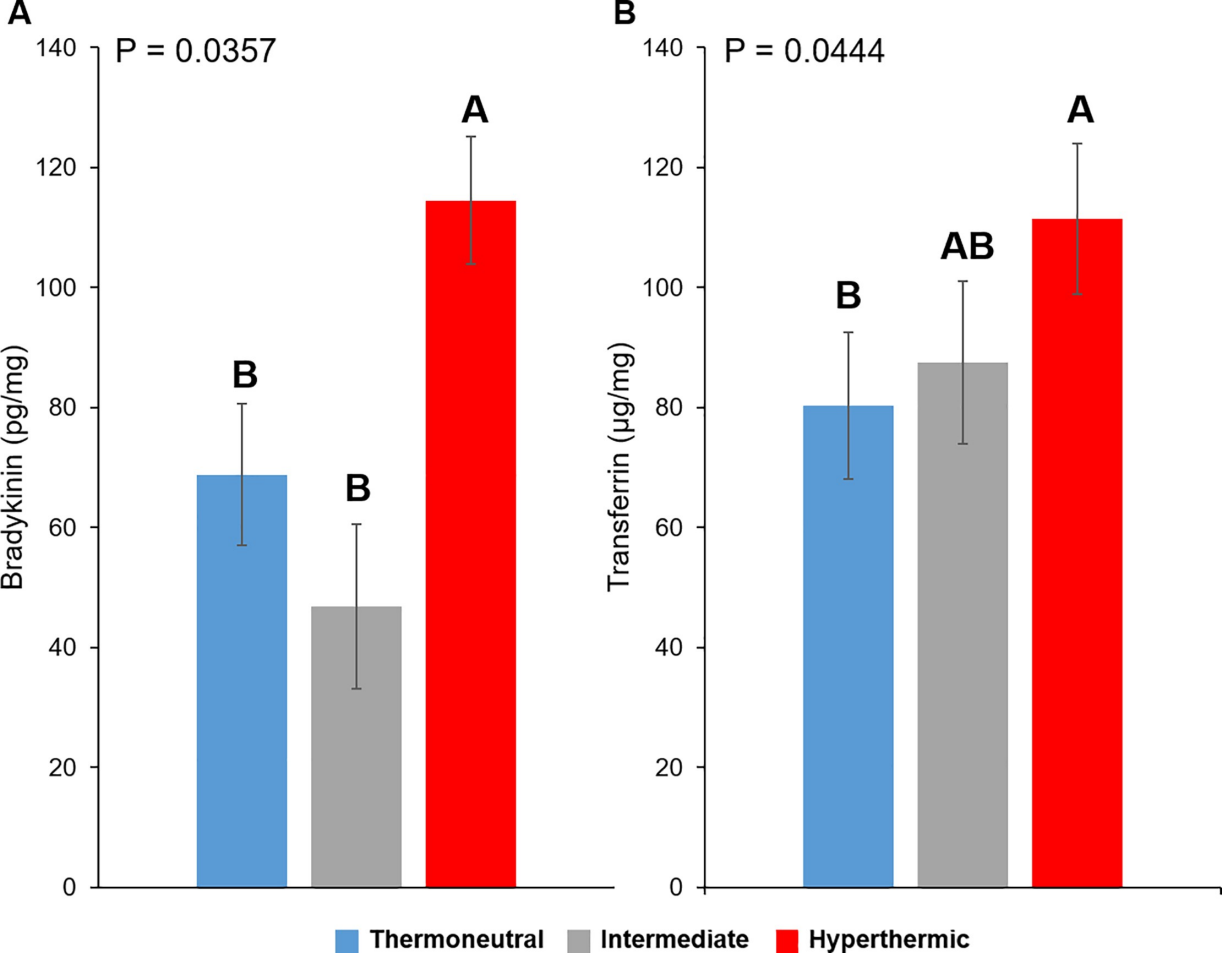
Levels of bradykinin and transferrin within follicular fluid from thermoneutral, intermediate and hyperthermic cows. Intrafollicular levels of bradykinin (pg per mg total protein; Panel A) and transferrin (μg per mg total protein; Panel B). ^AB^Bars (least squares means ± SEM) that do not share a letter differ significantly (P ≤ 0.05).

Intrafollicular levels of bradykinin and transferrin differed depending on whether cows became hyperthermic or exhibited an intermediate response to heat stress (P = 0.0357 and P = 0.0444, respectively; Fig 5). Cows classified as hyperthermic had higher intrafollicular levels of bradykinin than thermoneutral (P = 0.0456) or intermediate cows (P = 0.0175; Fig 5A). Concentrations of transferrin were higher in follicular fluid from hyperthermic cows compared to their thermoneutral counterparts (P = 0.0181) whereas cows classified as intermediate had intrafollicular levels that were between those obtained from thermoneutral and hyperthermic cows (P > 0.09; Fig 5B).

**Fig 6:**
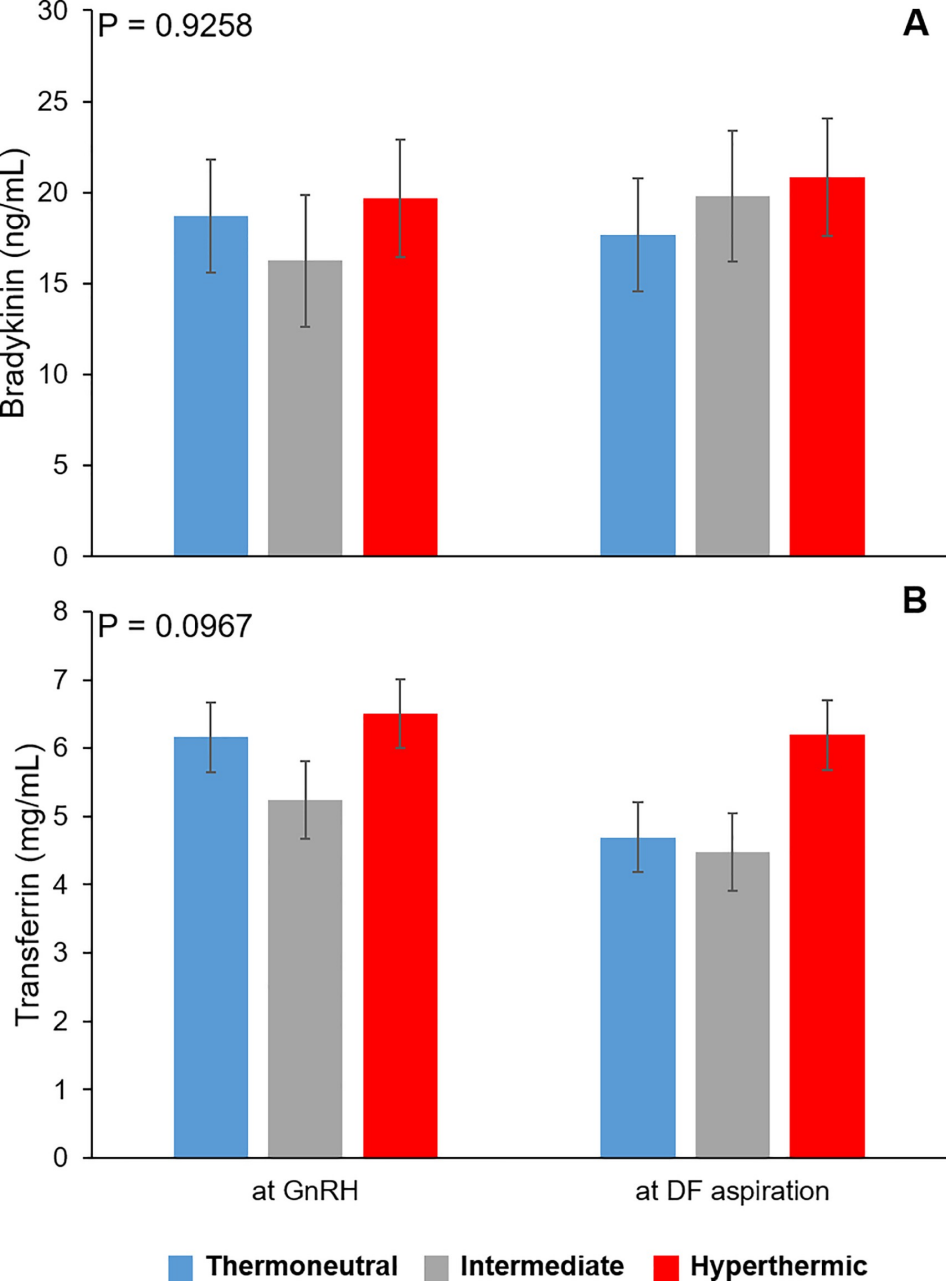
Circulating levels of bradykinin and transferrin within thermoneutral, intermediate and hyperthermic cows. Levels of bradykinin (A) and transferrin (B) in serum collected at GnRH administration but before environmental treatments were applied to cows and at time of dominant follicle (DF) aspiration (~16 h after GnRH administration).

Levels of bradykinin within the sera collected from thermoneutral, intermediate and hyperthermic cows did not differ at time of GnRH administration to induce LH surge (0 h of heat stress conditions) or dominant follicle aspiration (P = 0.9258; Fig 6A). Serum concentrations of transferrin tended to differ between groups depending on the time of sampling (P = 0.0967; Fig 6B). At initiation of heat stress conditions, transferrin levels within serum was similar between thermoneutral, intermediate and hyperthermic cows (P > 0.1). At the time of dominant follicle aspiration (~16 h post GnRH administration), amounts of transferrin tended to be greater in sera from hyperthermic cows compared to thermoneutral (P = 0.0683) and intermediate cows (P = 0.0570).

### Cytokines in follicular fluid and sera

Intrafollicular concentrations of cytokines CCL2, CCL4, IL-1β, IL-RA, Il-2, IL-4, IL-8, IL-10, IL-17A, IFNγ, and IP-10 were similar between thermoneutral, intermediate and hyperthermic cows (P > 0.2; Table 7). However, interleukin 6 (IL-6) concentrations were higher in follicular fluid from heat stressed animals (intermediate and hyperthermic) compared to thermoneutral counterparts (P = 0.0160; Table 7). Levels of CCL3, IL-1α, IL-2, IL-8, IL-10 and TNFα were at or below the level of detection in all aspirates of follicular fluid (Table 2). At the time of GnRH administration circulating cytokines levels were similar in cows that had been randomly allocated to be maintained under thermoneutral or heat stress conditions (P > 0.2; Table 8). Levels of circulating cytokines remained similar in thermoneutral and hyperthermic cows 16 h after GnRH administration (i.e., time of dominant follicle aspiration) (P > 0.2; Table 8).

**Table 7:** Levels of cytokines within follicular fluid aspirates.

Cytokine	Thermoneutral (pg/mg^1^)		Intermediate (pg/mg^1^)		Hyperthermic (pg/mg^1^)		P-Value
C-C motif chemokine ligand 2 (CCL2)	16.65	± 1.49	13.96	± 1.72	17.05	± 1.49	0.4529
C-C motif chemokine ligand 4 (CCL4)	0.25	± 0.19	0.46	± 0.24	0.82	± 0.21	0.2182
Interleukin 1β (IL-1β)	0.23	± 0.07	0.22	± 0.08	0.07	± 0.07	0.2773
Interleukin 1 receptor antagonist (IL-1RA)	3.04	± 1.53	4.03	± 2.01	2.08	± 1.59	0.7446
Interleukin 4 (IL-4)	2.52	± 1.14	5.65	± 1.33	4.64	± 1.20	0.2217
**Interleukin 6 (IL-6)**	**0.13**	**± 0.05**^**b**^	**0.46**	**± 0.06**^**a**^	**0.32**	**± 0.05**^**a**^	**0.0160**
Interleukin 17A (IL-17A)	0.01	± 0.002	0.01	± 0.002	0.01	± 0.002	0.9580
Interferon γ (IFNγ)	0.05	± 0.02	0.08	± 0.03	0.06	± 0.03	0.7021
Interferon γ-induced protein 10 (IP-10)	14.60	± 3.12	25.78	± 4.03	18.55	± 3.12	0.1851

^1^Cytokine values normalized by total protein concentration

^ab^Least squares means (± SEM) that do not share a letter differ significantly, cytokine and associated values with a significant P-value denoted in bold

**Table 8:** Levels of circulating cytokines.

	At GnRH to induce LH surge					16 h after GnRH^1^				
Cytokine	Thermoneutral(pg/mL)		Hyperthermic (pg/mL)		P-Value^2^	Thermoneutral (pg/mL)		Hyperthermic(pg/mL)		P-Value^3^
CCL2	621.9	± 182	767.0	± 189	0.5158	541.1	± 200	723.6	± 189	0.4544
CCL3	522.6	± 154	811.6	± 160	0.1522	652.5	± 170	890.6	± 160	0.2679
CCL4	146.3	± 21	89.5	± 24	0.0912	101.6	± 26	108.7	± 24	0.8357
IL-1α	102.6	± 55	150.3	± 57	0.4935	141.7	± 60	184.8	± 57	0.5676
IL-1β	45.0	± 33	50.7	± 33	0.8557	62.3	± 35	49.3	± 33	0.7083
IL-1RA	59.9	± 28	42.8	± 29	0.6720	89.9	± 33	103.5	± 29	0.7562
IL-2*	4.0	± 2.2	6.1	± 2.3	0.4106	5.3	± 2.4	7.3	± 2.3	0.4656
IL-4	81.9	± 44	48.0	± 47	0.4544	92.4	± 47	50.3	± 47	0.3993
IL-6	7.1	± 3.4	7.3	± 3.7	0.9741	9.5	± 3.7	13.0	± 3.9	0.5286
IL-8*	3.3	± 1.2	2.6	± 1.3	0.7143	2.1	± 1.3	2.3	± 1.2	0.9241
IL-10	296.0	± 218	560.5	± 225	0.3881	381.2	± 244	737.1	± 225	0.2836
IL-17A	0.8	± 0.6	0.9	± 0.6	0.8155	0.9	± 0.6	1.0	± 0.6	0.8754
IFNγ	1.1	± 0.3	1.5	± 0.3	0.1380	1.0	± 0.3	1.2	± 0.3	0.3770
IP-10	485.2	± 112	553.1	± 116	0.5526	489.5	± 121	569.6	± 116	0.5244
TNFα*	1.3	± 1.0	3.1	± 1.1	0.2224	2.2	± 1.2	3.8	± 1.1	0.2900

*Indicates concentrations at ng/mL

^1^Time of dominant follicle aspiration

Compared values from thermoneutral to hyperthermic cows at GnRH administration but before environmental treatments were applied to cows^2^

and at dominant follicle aspiration^3^.

Data presented as least squares means ± SEM.

## Discussion

When heat stress conditions were sufficient to induce hyperthermia in lactating dairy cows after an LH surge to induce ovulation some 28 h thereafter [35], impacts on the size of the ovulatory follicle and steroidogenic environment contained therein were minimal when examined approximately 16 h after administering GnRH. Efforts to take a closer look at the intrafollicular proteome and the abundance of certain proteins and cytokines however, revealed hyperthermia-related consequences which may be impactful on the continued progression of the follicle towards ovulation and on the developmental competence of the cumulus-oocyte resident within.

**Fig 7:**
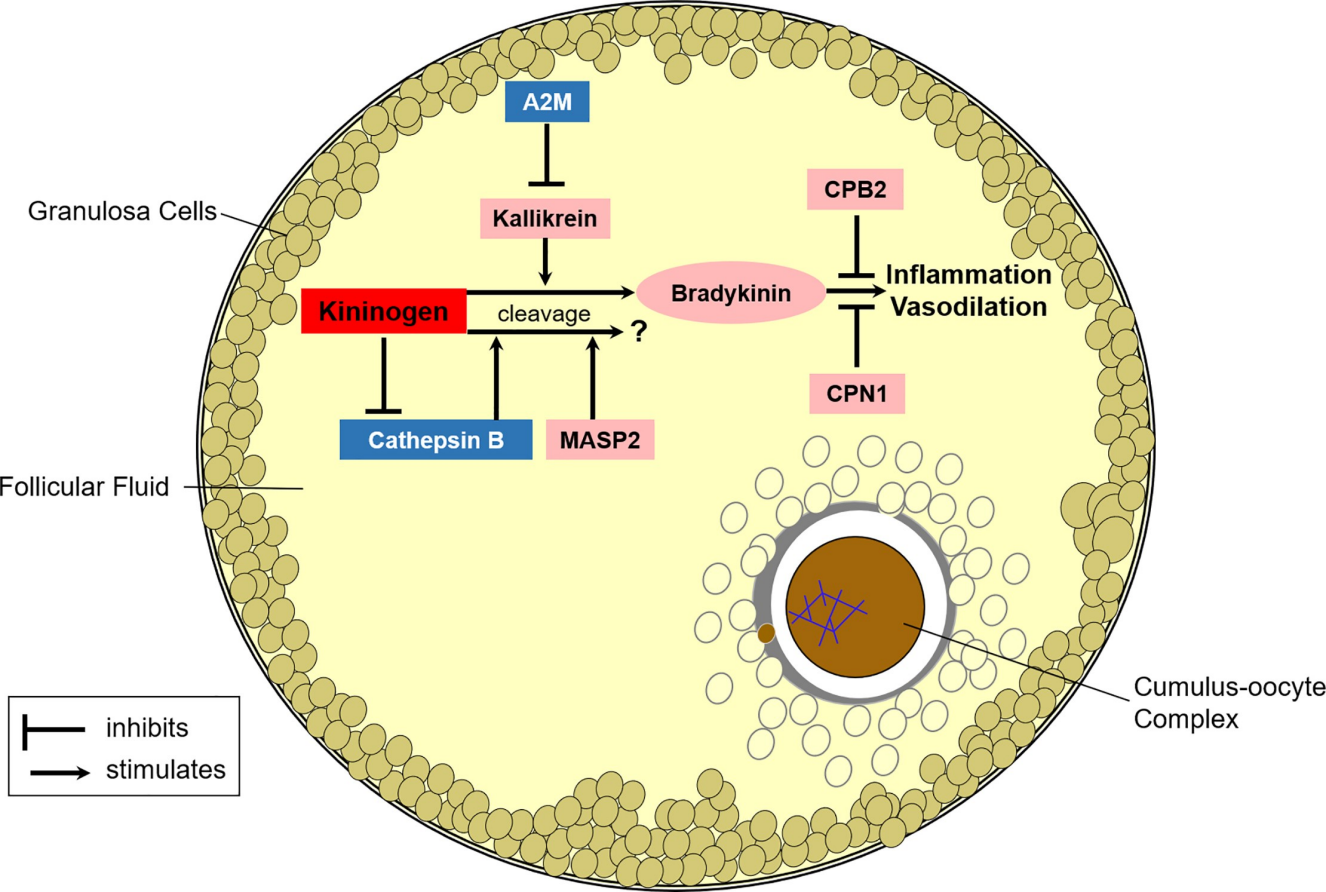
Simplified depiction of hyperthermia impacts on the intrafollicular complement and coagulation cascade. The effects of hyperthermia on protein levels within follicular fluid designated with color; red for greater than 2-fold increase in abundance, pink for moderate increases (1.2 to 1.7-fold) in abundance, and blue for decreases (1.6 to 2-fold) in abundance. Bradykinin peptide is released from precursor kininogen through proteolysis mediated by kallikrein enzyme [45]. The proinflammatory actions of bradykinin are inhibited by plasma metalloproteases carboxypeptidase N catalytic chain 1 (CPN1; [46]) and carboxypeptidase B2 (CPB2; [47]). Kininogen can also serve as a substrate for cathepsin B or mannan binding lectin serine peptidase 2 (MASP2), though proteolysis with either enzyme does not release bradykinin [48, 49]. In addition, kininogen can function as a weak inhibitor of cathepsin B [49]. Alpha-2-Macroglobulin (A2M) is an anti-proteinase that complexes with proteinases such as kallikrein [50] to inhibit proteolytic activity.

Hyperthermia-related impacts on those functionally annotated to the complement and coagulation cascade in the follicular fluid were especially notable (Fig 7) and are consistent with Min *et al*. [36] documenting shifts occurring in the plasma of chronically heat-stressed lactating dairy cows. Unique to our study were heat-induced effects on the kininogen-kallikrein system. Notably, hyperthermia induced an ~15-fold increase in abundance of intrafollicular kininogen. Very few studies have focused on kininogen protein levels within the ovary after gonadotropin stimulation [37, 38] and none could be located investigating the response to heat-induced elevations in body temperatures.

Kininogen is primarily defined as a precursor for kinin peptides, which mediate inflammation, vasodilation and prostaglandin synthesis, all of which are important to set the stage for ovulation (reviewed by [39–41]). The non-kinin portions of the protein (i.e., heavy and light chains) can have additional roles such as inhibiting cathepsins (Fig 7). Consistent with observations in the rat ovary [37], increased kininogen levels in our study were coincident with lower levels of cathepsin B. These consequences likely originated from effects on the follicle. Granulosa cells from Graafian follicles express the mRNA [42] and protein [43] for kininogen. In an *in vitro* study imposing elevated temperatures during oocyte maturation cathepsin B activity and protein levels increased in maturing bovine oocytes and their associated cumulus cells [44].

Bradykinin peptide levels within the follicular fluid of the hyperthermic cows in our study were not increased to a similar degree as observed for kininogen precursor (Fig 7). This may be because hyperthermia induced only a moderate increase in levels of the kallikrein enzyme necessary to cleave the bradykinin peptide from kininogen molecule. Ilha *et al*. [42] reported that GnRH-induced changes in intrafollicular bradykinin levels did not necessarily correspond to alterations in kininogen transcript expression in granulosa cells.

We cannot rule out the possibility that the moderate increases observed herein in carboxypeptidase N and B2 (Fig 7), known degrading enzymes of bradykinin [46, 47], may have modulated the bradykinin levels within follicular fluid. Elevations in bradykinin were only observed in the follicular fluid from hyperthermic cows indicating that the perturbation to the intrafollicular kininogen-kallikrein system may be temperature sensitive. In the rat and rabbit, bradykinin has been shown to potentiate follicular rupture [51, 52]. Specific to this end, bradykinin induced ovulation in the absence of gonadotropin but did not induce maturation of rabbit oocytes [51]. It remains unclear what consequence, if any, elevated intrafollicular levels of bradykinin may have on ovulation potential in mono-ovulatory cows that become hyperthermic after an acute heat stress event. Heat-stressed cows appear to ovulate without issue under chronic [53, 54] or acute conditions severe enough to induce hyperthermia at levels at or exceeding 41 C [8].

Transferrin emerged as another protein of interest not only because intrafollicular levels increased more than 2-fold in hyperthermic cows, but also because intrafollicular levels at time of oocyte retrieval in humans have been related to developmental competence of the oocyte [55]. This iron-binding glycoprotein, produced primarily by the liver, can be secreted locally into the follicular fluid by cumulus and mural granulosa cells [56]. Transferrin levels were lowest in fluid from follicles containing immature oocytes and ~10-fold higher in those with mature oocytes [55]. Related to follicles yielding matured oocytes, highest transferrin levels were associated with fertilization or cleavage failure [55]. Consequences of heat-induced elevations in transferrin on the cumulus-oocyte complex within the follicle of hyperthermic cows remain unclear because heat-induced reductions in fertilization or cleavage rates are seldom problematic [11, 12, 57].

Although underlying mechanisms remain unclear, intrafollicular changes in kininogen and transferrin levels in hyperthermic cows may be related to heat-induced changes in concentrations of cytokines within the follicle. Cytokines IL-6, IL-1α, IL-1β, and TNFα have been previously shown to upregulate transferrin in rodent Sertoli cells [58, 59] and the kininogen gene may have response elements for IL-6 and TNFα [60]. At the same time, kininogen cleavage products can stimulate cytokine secretion. Human mononuclear cells stimulate release of cytokines IL-1β, IL-6, IL-8, CCL2 and TNFα in response to the non-kinin portion of kininogen, but not bradykinin [61].

Of the nine cytokines detected in our study, only one (i.e., IL-6) was shown to be differentially abundant (increased) in the follicular fluid of heat-stressed cows. Interleukin 6 is a multifunctional cytokine having a role not only in inflammation and infection response, but also in regulation of metabolic, neural and reproductive processes [62]. Similar to transferrin, levels of IL-6 are higher in fluid from human follicles containing mature oocytes compared to those with immature oocytes [63]. Intrafollicular levels of IL-6 have been associated with pregnancy success after IVF and embryo transfer [64, 65]. *In vitro* studies with murine and bovine cumulus-oocyte complexes have shown that elevations in IL-6 promote cumulus expansion and meiotic progression during oocyte maturation [66, 67]. Whether or not levels of transferrin and/or IL-6 observed in our studies in the follicular fluid of hyperthermic cows impact developmental competence of the cumulus-oocyte complex remains to be determined.

Reproduction-relevant changes in intrafollicular IL-6 levels appear to be confined to the follicle and not reflected in the circulation [68]. This may explain why we observed significant increases in IL-6 in the follicular fluid of heat-stressed cows but not in the serum. The likely source of IL-6 within the follicle is the granulosa and theca cells. Increases in IL-6 mRNA expression occurs in granulosa and theca cells as the follicle transitions from estradiol active (E2:P4 > 1) to estradiol inactive (E2:P4 < 1) [69]. It is worth noting that the observed declines in numerous cytokeratins (KRT2, KRT3, KRT5, KRT10, KRT17, KRT18, and KRT75) within the follicular fluid may be further evidence of changes occurring within follicular cells of hyperthermic cows. Others have documented the presence of cytokeratins in follicular fluid [70–73]. Cytokeratins within follicular fluid could be attributed to granulosa cells releasing protein fragments as part of cell death [74] and/or after disintegration of cytokeratin filaments during gonadotropin-induced differentiation [75, 76]; both events are likely essential as the follicle reorganizes in preparation for ovulation. The reduced intrafollicular levels of cytokeratins coupled with decreased laminin (LAMA1) and collagen (COL4A1) presence observed herein support the notion that hyperthermia resulting from an acute heat stress event may be hastening structural changes within the periovulatory follicle.

Intrafollicular events leading to ovulation are comparable to an inflammation response due to the numerous inflammatory-like changes occurring in the periovulatory follicle after the LH surge (reviewed by [39]). Exposure to heat stress sufficient to induce hyperthermia after the LH surge may be having consequences on the normal release of inflammation effectors associated with oocyte competence acquisition in the bovine [77]. Nonetheless, many of the proteins identified herein have also been identified in other studies investigating bovine follicular fluid components. Independent of environmental conditions, 142 of our identified proteins are in common with those reported by Ferrazza *et al*. [78]; 39 are common those reported by Zachut *et al*. [79]. Across studies, the overall characteristics of the protein profile (i.e., distribution of molecular weights and isoelectric points) were similar despite differences in experimental design and mass spectrometry approach between studies attesting to relevance of profiles reported herein. Efforts of others to demonstrate enrichment of follicular fluid proteins associated with immune responses, in particular the complement system [78, 79] attest to relevance of hyperthermia related outcomes. Whether hyperthermia-induced changes in the heat-stressed cow’s follicular fluid milieu reflect changes in mural granulosa, cumulus or other cell types’ secretions and/or transudative changes from circulation remains to be determined. Regardless of origin, changes in the follicular fluid milieu may have an impact on components important for ovulation and competence of the cumulus-oocyte complex contained within the periovulatory follicle.

## Supporting information

S1 Fig Profiles for the size of the dominant follicle and hormones from the cows utilized in study. (TIF)

S1 File Proteins identified within periovulatory follicular fluid. (XLSX)
