# Supplementary figures and images for: Heat-induced hyperthermia impacts the follicular fluid proteome of the periovulatory follicle in lactating dairy cows
Source: PLoS One. 2019 Dec 30;14(12):e0227095. doi: 10.1371/journal.pone.0227095 (PMC6936800; doi:10.1371/journal.pone.0227095)

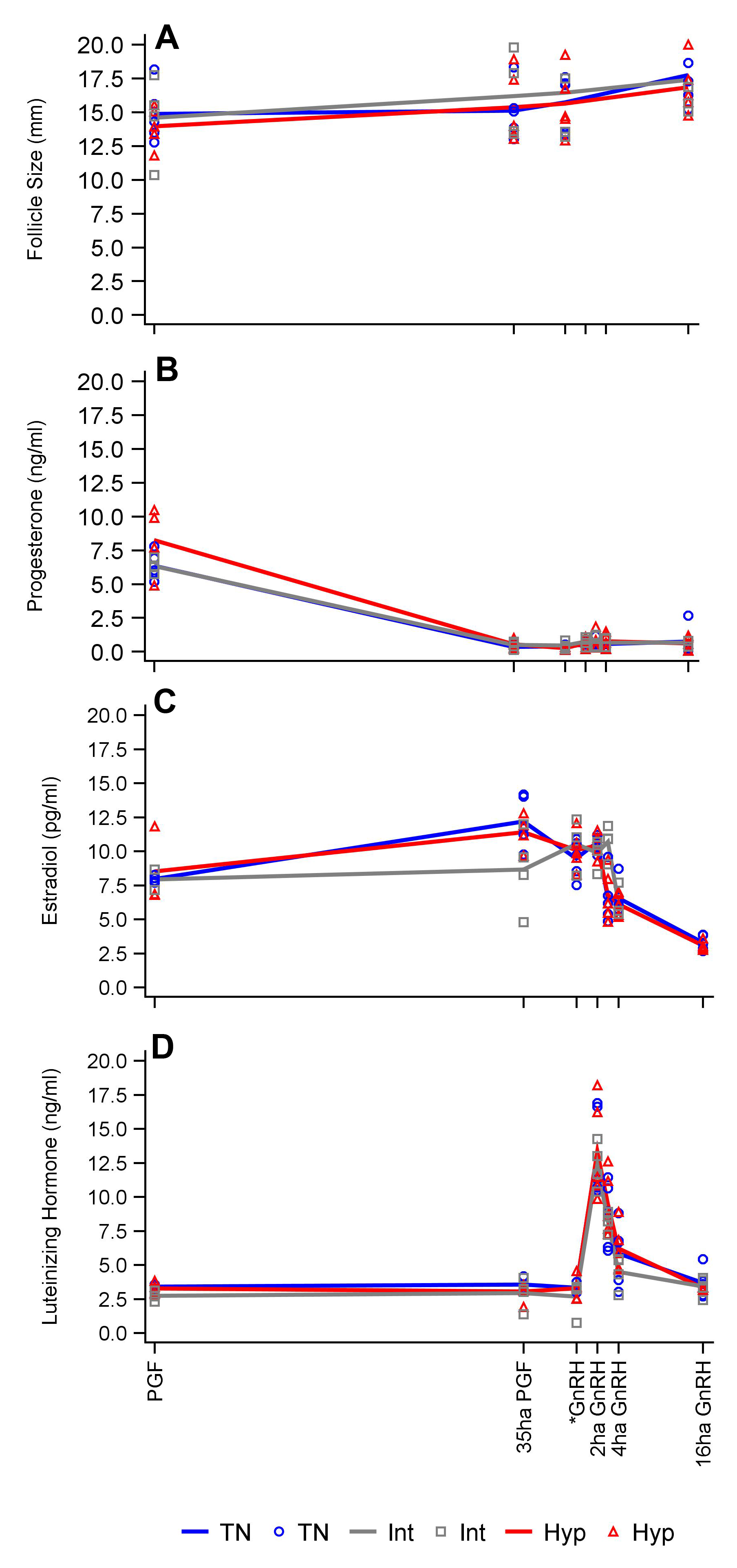

Supplement: S1 Fig — (TIF) [file pone.0227095.s001.tif]
